# Genome-wide identification and characterization of functionally relevant microsatellite markers from transcription factor genes of Tea (*Camellia sinensis* (L.) O. Kuntze)

**DOI:** 10.1038/s41598-021-03848-x

**Published:** 2022-01-07

**Authors:** Rajni Parmar, Romit Seth, Ram Kumar Sharma

**Affiliations:** 1 Biotechnology Department, CSIR-Institute of Himalayan Bioresource Technology (CSIR-IHBT), Palampur, Himachal Pradesh 176061 India; 2 Academy of Scientific and Innovative Research (AcSIR), CSIR-HRDC Campus, Ghaziabad, Uttar Pradesh 201 002 India

**Keywords:** Biotechnology, Computational biology and bioinformatics, Genetics, Molecular biology, Plant sciences

## Abstract

Tea, being one of the most popular beverages requires large set of molecular markers for genetic improvement of quality, yield and stress tolerance. Identification of functionally relevant microsatellite or simple sequence repeat (SSR) marker resources from regulatory “Transcription factor (TF) genes” can be potential targets to expedite molecular breeding efforts. In current study, 2776 transcripts encoding TFs harbouring 3687 SSR loci yielding 1843 flanking markers were identified from traits specific transcriptome resource of 20 popular tea cultivars. Of these, 689 functionally relevant SSR markers were successfully validated and assigned to 15 chromosomes (*Chr*) of CSS genome. Interestingly, 589 polymorphic markers including 403 core-set of TF-SSR markers amplified 2864 alleles in key TF families (bHLH, WRKY, MYB-related, C2H2, ERF, C3H, NAC, FAR1, MYB and G2-like). Their significant network interactions with key genes corresponding to aroma, quality and stress tolerance suggests their potential implications in traits dissection. Furthermore, single amino acid repeat reiteration in CDS revealed presence of favoured and hydrophobic amino acids. Successful deployment of markers for genetic diversity characterization of 135 popular tea cultivars and segregation in bi-parental population suggests their wider utility in high-throughput genotyping studies in tea.

## Introduction

Microsatellites or Simple Sequence Repeats (SSRs) are ubiquitously abundant variable tandem DNA repeats (1-6 bp) generated due to replication slippage and DNA repair mechanism in both prokaryotic and eukaryotic genomes^1,2^. Contrary to decades old belief of junk DNA, SSRs significance has been reported in chromatin organisation, gene function and DNA metabolic processes^3^. SSR markers particularly derived from functionally characterised protein coding and non-coding un-translated regions (UTRs) are categorised as “functional markers”. Additionally, good marker attributes of SSRs over other technologies suggests their wider utility in fingerprinting, diversity characterization, evolutionary, genome mapping and comparative genomic studies^4–6^.

Tea (*Camellia sinensis* (L) O. Kuntze), due to its ability to accumulate about 700 medicinally important bioactive ingredient including phenolic (18–36%), amino acid (1–4%) and volatile compounds (0.03%), is one of the most popular non-alcoholic beverage, worldwide^7,8^. Being second largest global producer and custodian of highly heterogeneous germplasm resources with unique flavour, aroma and taste, “The Indian Hybrid Tea” is witness of successful commercial cultivation of tea in many developing countries including Kenya and Sri Lanka^9–11^. Nevertheless, negative impact of climate change induced extreme weather conditions are affecting the quality attributes, growth, yield and stress tolerance of tea, globally^12^. Therefore, there is an urgent need for breeding climate resilient high yielding quality tea cultivars for commercial cultivation. The conventional breeding approaches are offsets due to several bottlenecks i.e. perennial nature, self-incompatibility, heterozygosity and long breeding cycle of tea. Therefore, creation of functionally relevant novel SSR marker resource can assist in rapid identification of key QTLs, and expediting breeding of superior tea cultivars. Furthermore, cultural practices including monoculture/clonal cultivation of commercial tea plantations will have larger implications of novel core-set of SSR markers in developing unique DNA fingerprints for testing varietal/cultivars purity, authentication of potential tea cultivars or clones and various teas in global market^13^. Furthermore, multiple attributes such as multi-allelic nature, co-dominant inheritance, hyper-variability, chromosome-specific location, ubiquitous occurrence, high polymorphic information content (PIC) and reproducibility, TF derived novel SSR markers identified in this study can be potentially utilized for genetic improvement of tea^14,15^.

Dissection of underlying mechanism of desirable complex traits are challenging due to highly regulated structural gene networks^16^. Being ‘master regulator’ of various cellular processes, TF genes can be an excellent target for identification of functionally relevant SSR markers having greater implications in molecular dissection of complex traits in tea. Earlier studies have reported the *Teosinte branced1* (*Tb1*) of TCP TF family in maize and *qSH1* TFs responsible for lower rice grain shattering in domestication of maize and rice^17,18^. Furthermore, TFs genes with well-characterised functional domain harbouring polymorphic SSRs markers (expansion/contraction) possibly affecting the gene function can assist in rapid identification of key QTLs in tea. Interestingly, cost effective next generation global transcriptome sequencing offers greater opportunity in rapid elucidation of underlying regulatory networks of diverse agronomic traits and creation of genome-wide functionally relevant marker resources^19^.

In the present study, successful efforts were made for the first time to identify transcription factors (TFs) derived-SSR markers in tea. Functionally relevant marker resource comprising of 1843 novel TF-SSR markers exhibiting genome-wide representation across all 15 chromosomes^28^ was developed by using trait-specific (yield, quality and biotic/abiotic stress) in-house transcriptome data of 20 popular tea cultivars. Furthermore, the protein–protein interaction, gene ontology and localization (CDS & UTRs) identified functional relevance of novel TF- SSR markers in trait dissection. Constraints of existing SSR markers resources due to limited availability of experimentally validated SSR markers (~ 2000s)^14,15,20–24^, the identification of 589 polymorphic novel markers including 403 core-set of the TF-SSRs in the current study will be an excellent asset for various genotyping studies in tea^13,25–27^. Successful extrapolation of informative core-set of markers for genetic diversity assessment of 135 popular tea cultivars and expected segregation patterns in bi-parental mapping population suggests the wider utility of novel TF-SSR marker resource in marker-trait association, genetic diversity and phylogenetic studies in tea^20^.

## Results

### Frequency and distribution of SSRs

**Figure 1:**
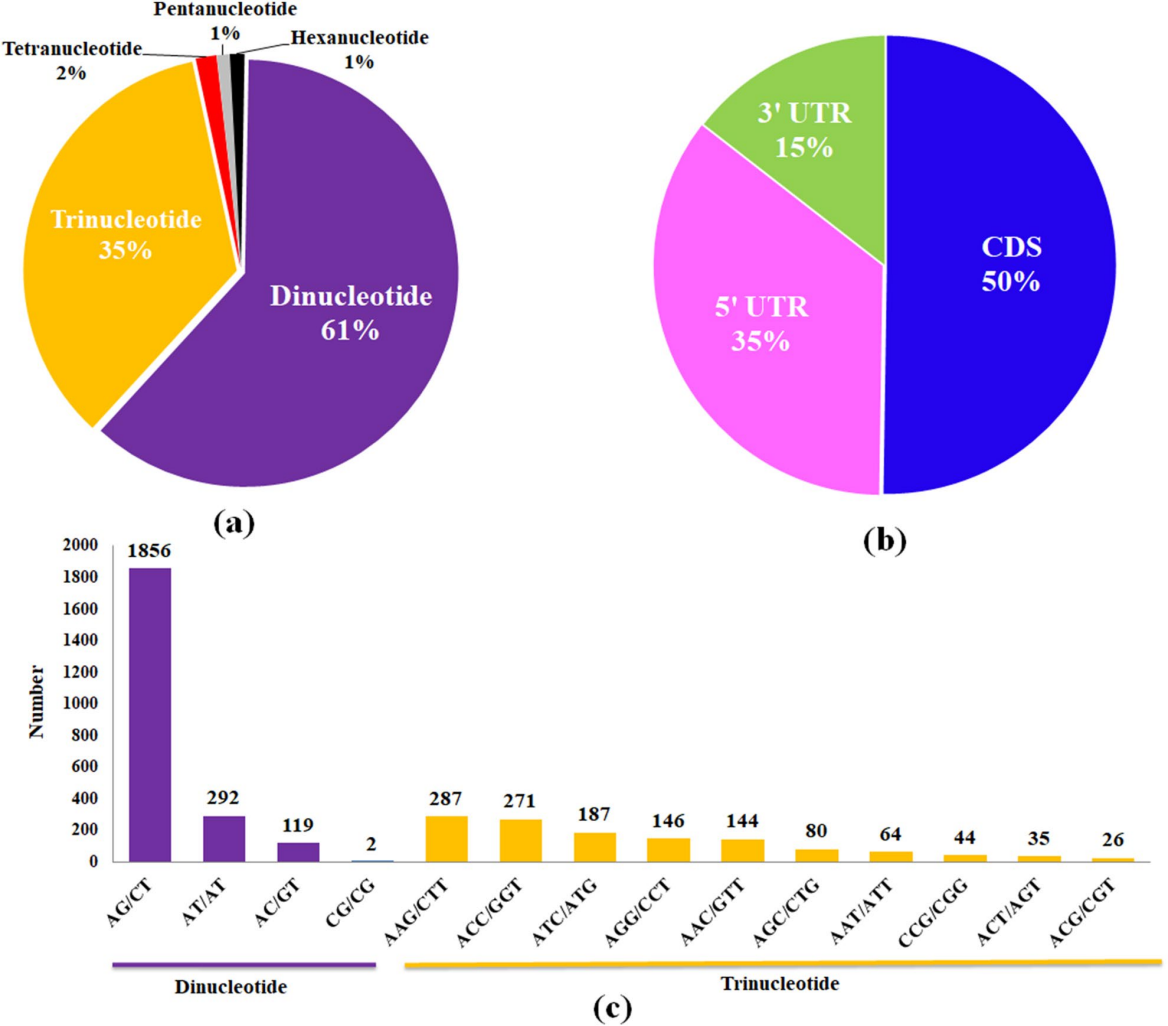
Abundance and localization of various SSRs transcription factors in tea: (**a**) Overall abundance of various SSR repeats; (**b**) Localisation of SSR repeats in CDS and UTRs; (**c**) Distribution frequency of major repeat motif types.

De novo assembly of high quality reads resulted into 194,558 non-redundant (NR) transcripts. Subsequently, microsatellites search identified 16,867 SSRs in 13,836 NR transcripts (Table 1). BLAST searched using Plant-TFIIdb retrieved 2776 TF encoding transcripts harbouring 3687 SSRs (Perfect: 3263; Compound repeats: 424) (Table 2). Overall, di-nucleotide SSRs repeats were most abundant (2269; 61.5%), followed by tri- (1284; 34.8%), tetra- (58; 1.57%), hexa- (41; 1.11%) and penta-nucleotide repeats (35; 0.94%) (Table 3; Fig. 1a).The shorter repeat motifs were more abundant with overall base composition bias towards As and Ts in the TF genes. Further, localisation identified presence of SSR repeats in CDS (50%), 5'UTR (35%) and 3'UTR (15%), respectively; wherein, tri-nucleotide repeats were more abundant in CDS (Fig. 1b). Among the di-nucleotide repeats, AG/CT, AT/TA, AC/GT were most prevalent. Similarly, tri-nucleotide (AAG/CTT, ACC/GGT, ATC/ATG, AGG/CCT, AAC/GTT), tetra-nucleotide (AAAG/CTTT, AAAT/ATTT, AGAT/ATCT), penta-nucleotide (AAAAG/CTTTT, AAAAC/GTTTT, AAAAT/ATTT and hexa-nucleotide repeats (AAAACC/GGTTTT, AACACC/GGTGTT, AACCAG/CTGGTT) were the most represented (Fig. 1c).

**Table 1:** Statistics of overall de novo assembled transcripts derived from transcriptome sequencing of twenty tea cultivars.

Search items	Number
Total number of sequences examined	194,558
Total size of examined sequences (bp)	80,606,425
Number of SSR containing sequences	13,836
Total number of identified SSRs	16,867
Number of sequences containing more than 1 SSR	2354
Number of SSRs present in compound formation	1739

**Table 2:** Statistics of SSRs mined from transcription factor encoding transcripts of Tea.

Search items	Number
Total number of sequences examined	2776
Total size of examined sequences (bp)	4,321,851
Number of SSR containing sequences	2776
Total number of identified SSRs	3687
Number of sequences containing more than 1 SSR	677
Number of SSRs present in perfect formation	3263
Number of SSRs present in compound formation	424

**Table 3:** Overall abundance of SSRs repeat motifs in transcripts encoding transcription factor genes of tea.

Unit size	Number of SSRs
Di-nucleotide Repeat	2269
Tri-nucleotide-Repeat	1284
Tetra-nucleotide-Repeat	58
Penta-nucleotide-Repeat	35
Hexa-nucleotide-Repeat	41

### Identification, distribution of SSRs in TF genes

**Figure 2:**
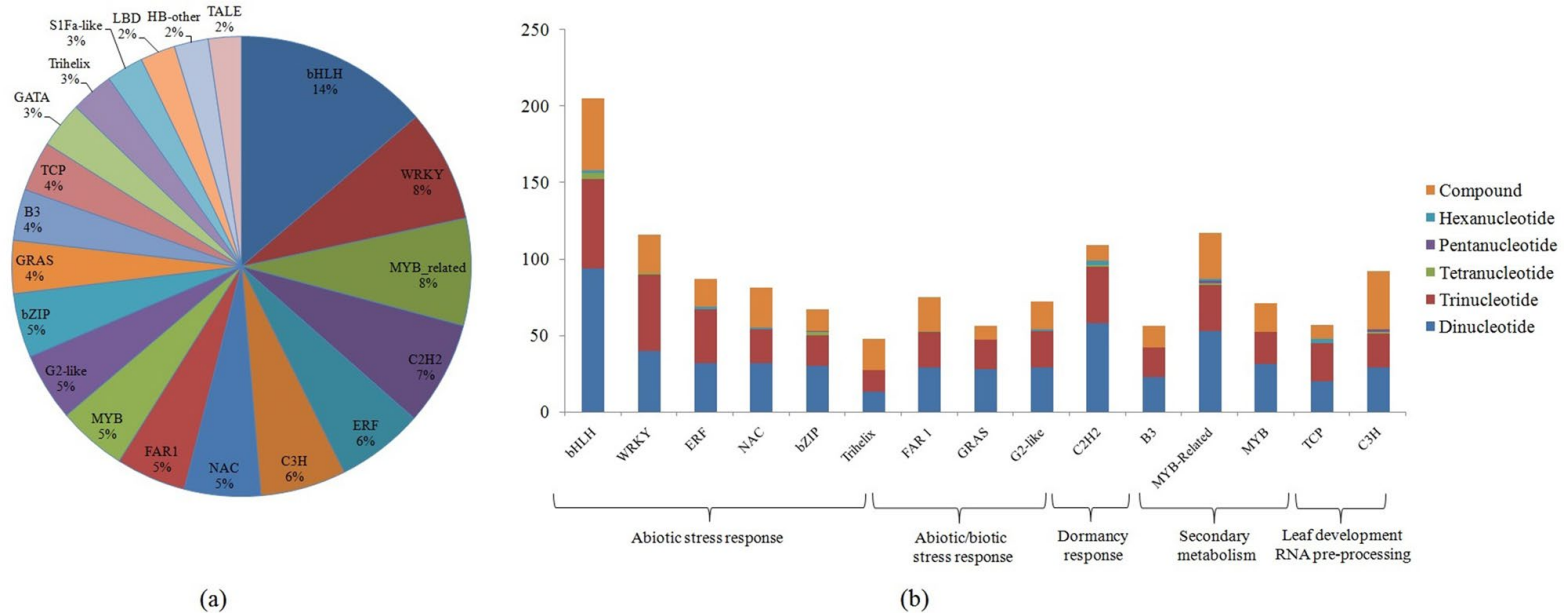
Details of transcription factors families harbouring SSRs in tea: (**a**) Top 20 SSR containing transcription factor families; (**b**) Distribution and frequency of various repeat types and functional relevance of major transcription factor families.

TFs control the physiological and regulatory networks of various functional genes to maintain the normal growth and response against various biotic and abiotic stresses in higher plants^28,29^. Approximately, 7.5% of the *Arabidopsis* genome encodes for TFs, clearly indicates their potential role in regulating diverse gene function^30^. In the current study, TF-SSRs recorded with higher abundance in bHLH (14%), followed by Myb-related (8%), WRKY (8%), C2H2 (7%), C3H (6%), ERF (6%), NAC (5%), FAR1 (5%), G2-like (5%) and MYB (5%) TF families (Fig. 2a). The di-and tri-nucleotide repeats were more abundant irrespective to TF families having key role in regulating abiotic & biotic stress response (bHLH, WRKY, ERF, NAC, bZIP, Trihelix, FAR1, GRAS, G2-like), secondary metabolism (MYB & MYB related), dormancy (C2H2, B3) and leaf development (TCP, C3H) (Fig. 2b). Furthermore, SSR marker loci harbouring bHLH (53), WRKY (37), C3H (32), Myb-related (31) and NAC (30) TF families exhibited with highly polymorphic and stable amplifications suggests their potential role in trait dissection. Majority of the TF families, such as, B3, bZIP, C2H2, C3H, G2-like, GRAS, MYB and WRKY contained AT-rich SSRs motifs. Moreover, bHLH, FAR1, Myb-related and NAC TF families contained both AT and GC rich SSRs, while only GATA TF family harbours GC-rich repeats^31^ (Fig. S1).

### Gene ontology analysis of SSRs containing TFs genes

**Figure 3:**
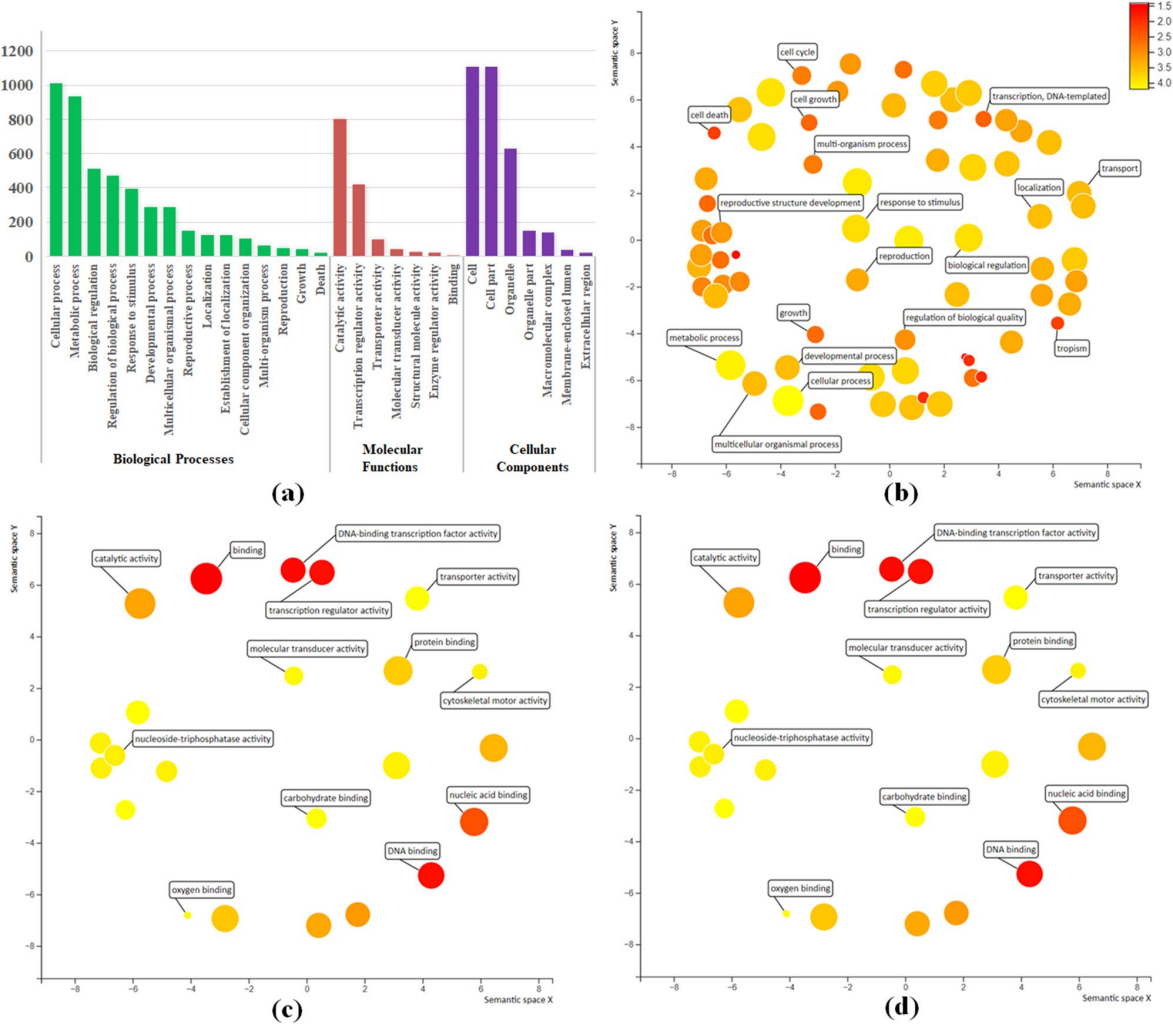
Gene Ontology (GO) analysis: (**a**) GO annotation of transcripts harbouring SSR loci’s classified into Biological Process, Cellular Component and Molecular Functions; (**b**) GO enrichment analysis representing Biological Process; (**c**) Cellular Component; (**d**) Molecular Functions.

Gene Ontology (GO) analysis was performed by significantly enriching the GO terms annotated Transcription Factor (TF) genes harbouring SSRs having significant homology with the well categorized predetermined *Arabidopsis thaliana* GO terms into respective biological processes, molecular functions and cellular components (Fig. 3a). In total, 7686 GO terms were assigned to 2128 transcripts categorized into biological process (4538 GO terms; 15 categories), molecular function (401 GO terms; 7 categories) and cellular component (3176 GO terms; 7 categories). Among the biological processes, biological regulations (GO:0065007), regulation of metabolic processes (GO:0019222) and regulation of gene expression (GO:0010468) were most represented followed by response to stimulus (GO:0050896), response to abiotic stress (GO: 0009628), response to metabolic processes (GO:0008152), cellular macromolecule biosynthesis processes (GO: 0034645) and transcription (GO: 0006350) (Fig. 3b). Likewise, among cellular components sub-categories including Cell (GO: 0005623), cell part (GO: 0044464), intracellular organelle (GO: 0043229) and nucleus (GO: 0005634) were the most abundant (Fig. 3c). Among the molecular functions, sub-categories namely transcription regulator activity (GO:0030528), transcription factor activity (GO: 0003700), catalytic activity (GO: 0003824), transferase activity (GO: 0016740) and kinase activity (GO: 0016301) were most represented (Fig. 3d). Overall, GO categorization suggests key role of SSR containing TFs genes in regulating complex metabolic pathways involved in yield, quality and stress (biotic & abiotic) tolerance (Fig. S2a-c).

### Transcription Factor derived MicroSatellite (TTFMS) markers

Of the 3687 SSR loci present in 2776 TFs, 1843 SSRs loci fulfilling the criteria of primer designing [GC content (40–60%), primer length (18–24 bp), estimated amplicon size (100-350 bp)] were successfully utilized for designing of flanking Tea Transcription Factor derived MicroSatellites (TTFMS) primer pairs. Further, localization of SSRs identified abundance in CDS (925) followed by un-translated 5'UTR (651) and 3'UTR (267) regions. Considering, importance in defining the characteristics, localization and key regulatory role of the transcription factor genes, functional domain may have greater implication in trait dissections^34^. Interestingly, 28 SSR Tea Transcription-factor derived Functional Domain MicroSatellite (TTFDMS) markers were identified in the functional domain of TF genes. The novelty of newly identified TTFMS and TTFDMS was established with cross referenced publicly available SSR markers resources in tea. The novel 1843 TF derived MicroSatellite markers resource (TTFMS, 1815; TTFDMS, 28) created for the first time in tea is available online at https://www.ihbt.res.in/en/miscellaneous/genomic-resources (Tea Transcription Factor Derived Microsatellite Resource).

### Evaluation of polymorphic potential

To evaluate the polymorphic potential, a panel of 862 functionally relevant TTFMS markers [CDS: 416; 5'UTR: 289 and 3'UTR: 129; functional domain (FD): 28], reportedly involved in the regulation of yield, quality and stress responsive genes were synthesized and experimentally validated in diverse tea cultivars. Of these, 589 markers exhibited with stable and robust polymorphic amplifications identifying 2864 alleles (Table 4). Allele numbers (*Na*) ranged from 2 to 17 per locus, while, mean value of polymorphic information content (PIC), gene diversity (*He*), observed heterozygosity (*Ho*) recorded was 0.60, 0.48, and 0.73, respectively (Supplementary file 1). Functional domain (FDs), being master regulator controlling various complex cellular processes, identification of 18 polymorphic TTFDMS markers stipulates their direct implications in trait dissection^32–34^. Furthermore, *PI* & *PIsib* statistics identified 403 core-set of markers with *PI* (< 0.2), *PIC* (≥ 0.5) and amplicon size difference of two alleles (> 3 bp), can be utilized for the larger-scale fingerprinting studies in tea (Supplementary file 2).

**Table 4:** Overall wet lab validation results of 862 TF-SSRmarkers of Tea.

Gene Region Targeted	Polymorphic	Monomorphic
CDS	297	56
5' UTR	211	30
3' UTR	81	14
Outcome	589	100

*173 TTFMS loci did not shown amplification.

### Genome-wide chromosomal assignment of TF-SSR markers

**Figure 4:**
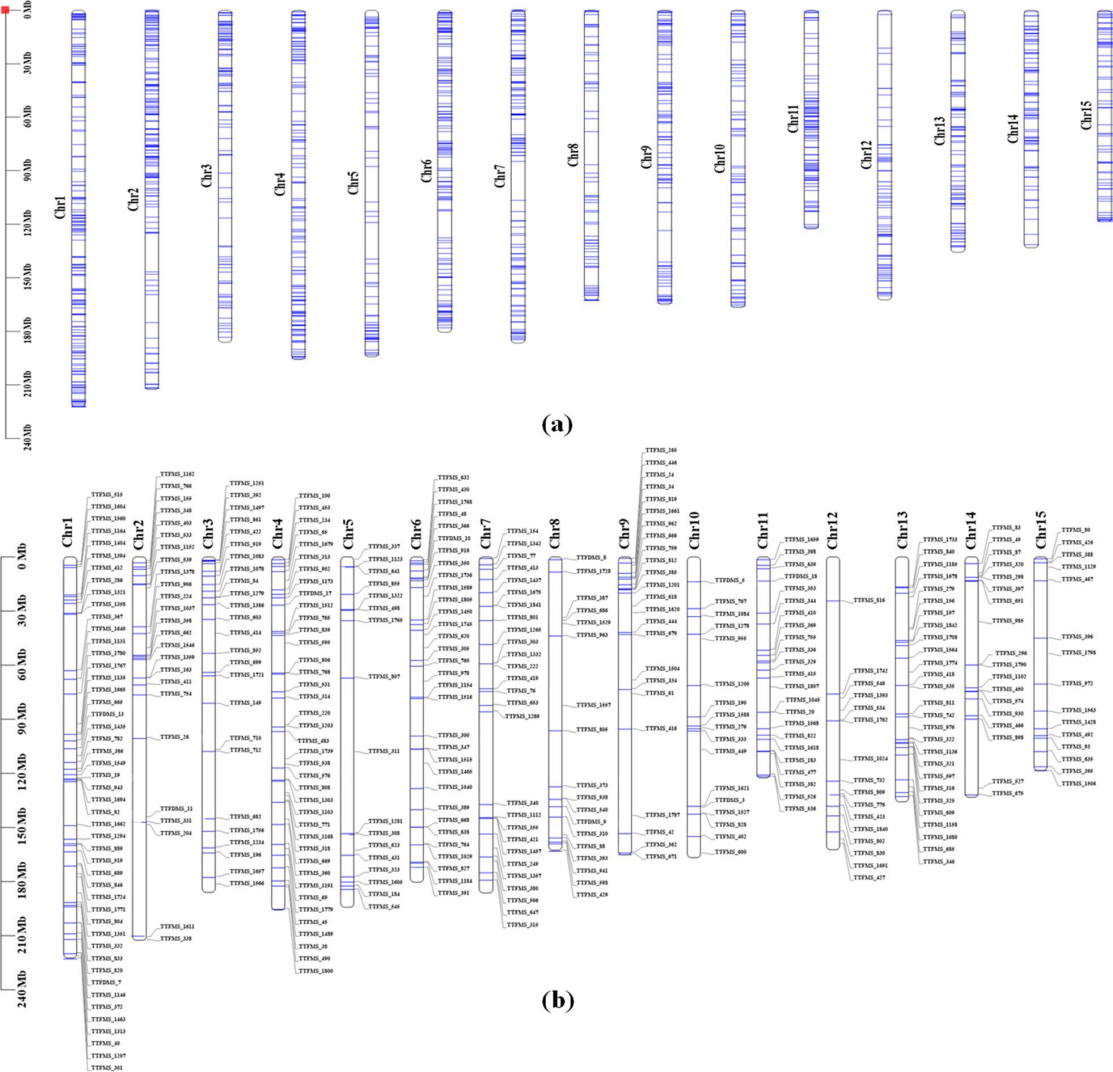
Chromosomal assignment of transcription factors derived SSR markers in tea CSS genome: (**a**) Overall assignment of SSR containing transcription factors to the 15 chromosomes; (**b**) Genome-wide assignment of experimentally validated polymorphic SSR markers to the 15 chromosomes of Tea.

Overall, 1977 transcripts encoding 55 transcription factor families harbouring SSR repeats were successfully mapped to 15 chromosomes of CSS tea genome^28^. Maximum transcripts were mapped to chromosome *Chr*1 (206), followed by *Chr*4 (187); *Chr* 2 (184); *Chr* 6 (175); *Chr* 7 (161); *Chr* 9 (129); *Chr* 3 (126); *Chr*11 (126); *Chr*13 (118); *Chr* 14 (110); *Chr* 10 (97); *Chr* 8 (95); *Chr* 5 (89); *Chr* 12 (87) and *Chr *15 (87). Likewise, experimentally validated SSR markers representing 47 TF families recorded genome-wide representation with majority of the potential polymorphic markers assigned to *Chr*4 (46); *Chr*7 (31); *Chr*1 (29); *Chr*2 (29); *Chr*11(27); *Chr*13 (22); *Chr*6 (21); *Chr*3 (20); *Chr*9 (20); *Chr*10 (18); *Chr*5 (17); *Chr*14 (17); *Chr*8 (16); *Chr*12 (12); *Chr*15 (15) and *Chr*12 (12) (Fig. 4). Interestingly, SSR containing TF families reportedly involved in key secondary metabolism attributing quality traits (bHLH, MYB, and MYB-related), biotic and abiotic stress responses (GRAS, NAC, WRKY, ERF, HSF and Tri-helix) and dormancy (B3, C2H2, and MYB) recorded wider distribution across 15 chromosomes of tea. Likewise, TF families reportedly involved in regulating bud/leaf colour and leaf development (Nin-like) were mapped to *Chr*2, *Chr* 3, *Chr* 7, *Chr* 8, *Chr* 11 and *Chr* 12.

### Protein–protein interaction (PPI) network

**Figure 5:**
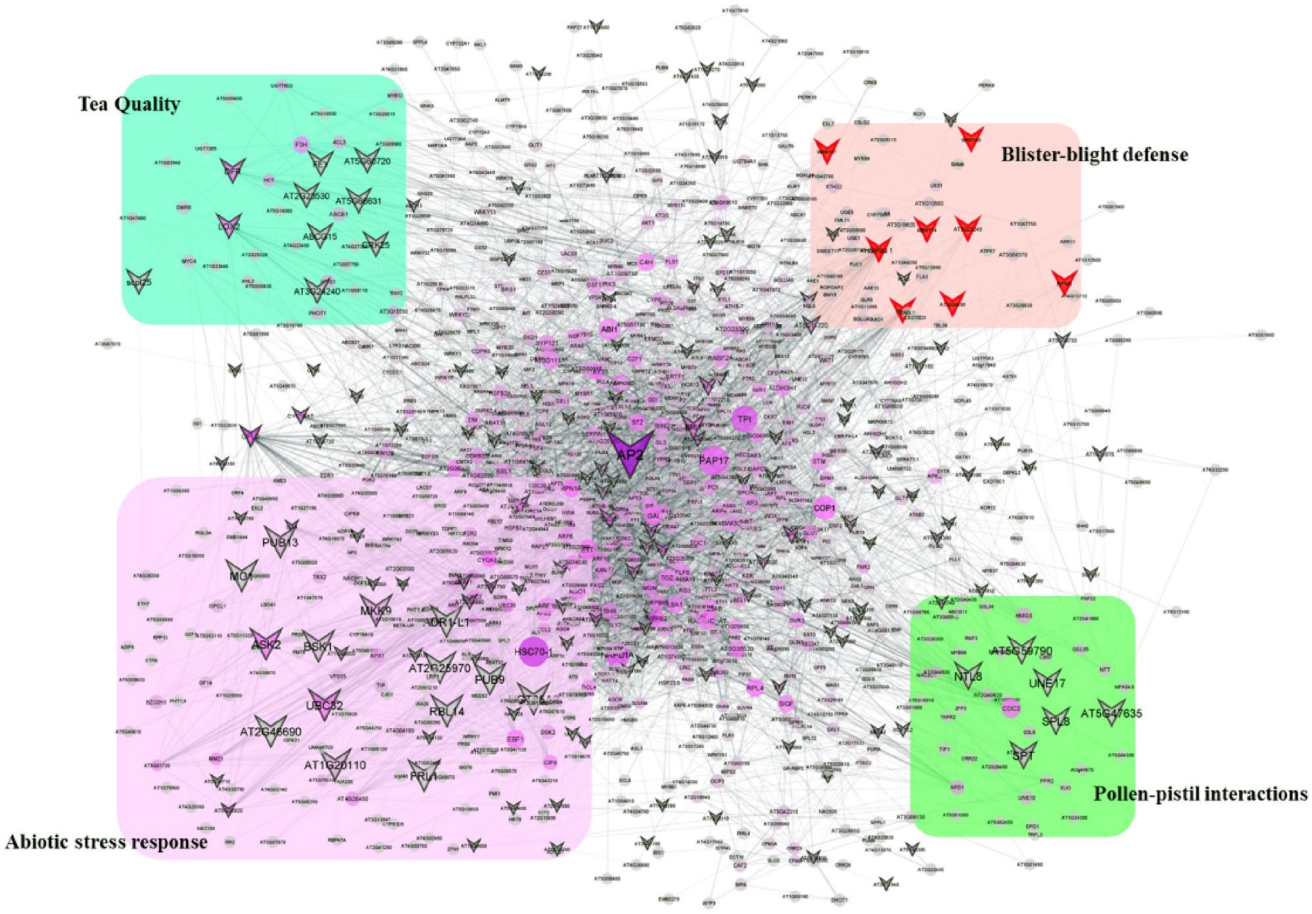
Protein–protein interaction (PPI) network analysis: PPIN prediction of transcription factors harbouring SSRs including polymorphic SSR markers using Cytoscape v3.4 (https://cytoscape.org).

Transcription factors act as a ‘switch’ regulating functional gene expression to maintain underlying mechanism of growth, development and stress responses (biotic/abiotic) in plants. The variations in the SSR repeats harboured by these TFs might influence the gene function affecting the phenotype. Therefore, interactome network analysis between the transcription factor genes containing SSRs and other functional proteins can assist in prediction of key trait specific markers regulating various signalling cascades. Overall, 1008 TF encoding transcripts were successfully mapped to predetermined PPI network of *A. thaliana,* interacting with 4779 proteins with average number of neighbours and clustering coefficient of 8.169 and 0.209, respectively (Fig. 5). Interestingly, TFs harbouring 170 polymorphic TF-SSR markers recorded with potential interactions, such as, WRKY exhibited interaction with LOX genes involved in biosynthesis of volatile fatty acids, while, NAC7 and bHLH93 were interacting with ATHB15 and DOF4.6 genes, reportedly involved in drought stress response and lignin biosynthesis, respectively. Likewise, ERF, MYB and AP2 were also found interacting with heat stress TFs^12^, cytokinin responsive growth, regulation of circadian rhythms regulators, response to blister blight defence and pollen-pistil interaction in tea (Fig. 5; Supplementary file 3).

### Genetic diversity analysis

**Figure 6:**
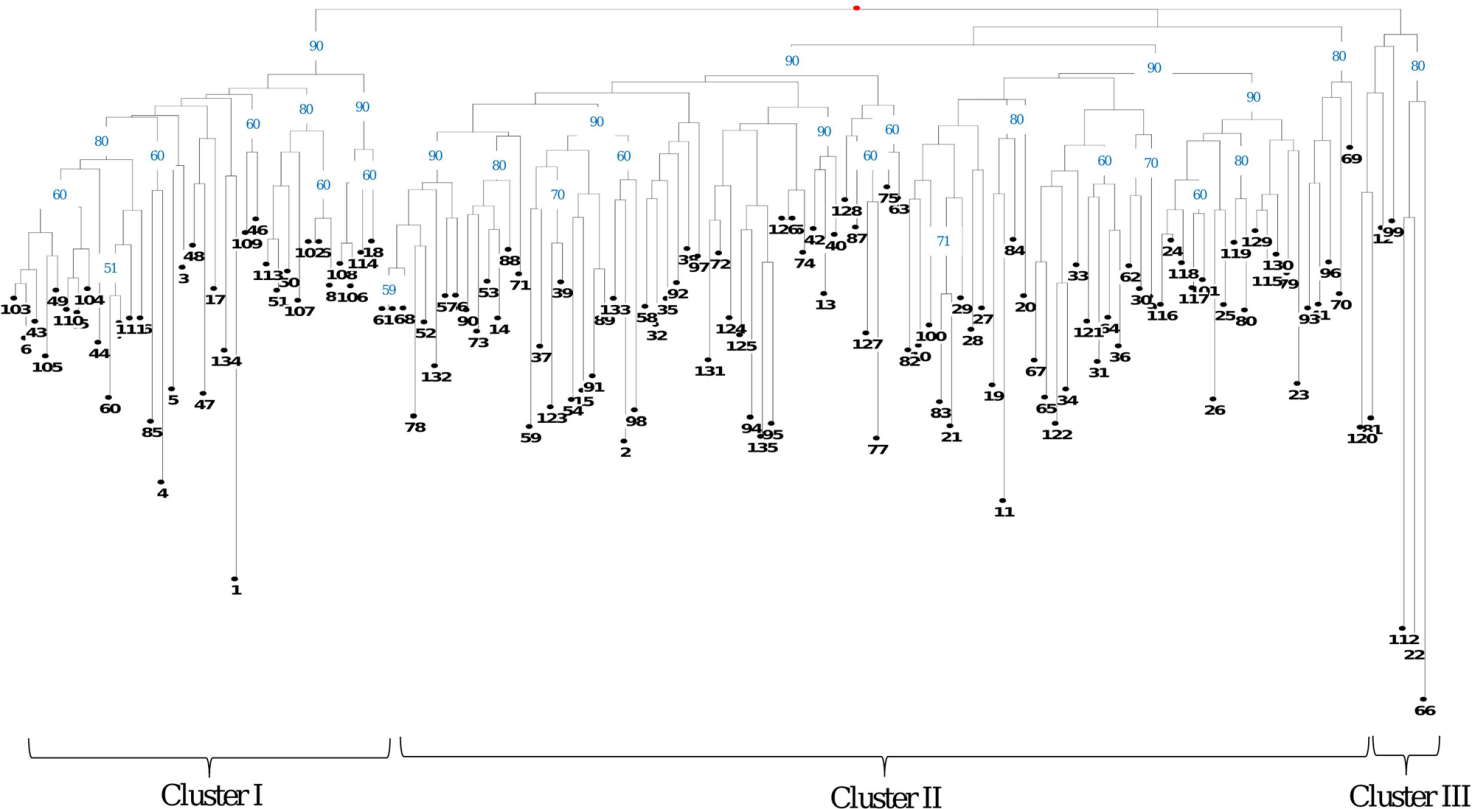
Cluster analysis: Neighbour-joining tree based on genetic distances of 135 tea cultivars using 15 polymorphic TF-SSR markers. Numbers above branches indicate bootstrap values ≥ 50% (1000 bootstrap replicates).

A subset of 15 core-set of polymorphic TTFMS markers derived from potential transcription factor families like bHLH, MYB, WRKY, C3H, ERF and FAR1 were utilized for diversity characterization of 135 popular tea cultivars representing collections of commercial tea estates, abandoned tea gardens of north-western Kangra region and elite tea clones maintained at CSIR-IHBT field gene bank^35^. Interestingly, all the cultivars were uniquely distinguished and grouped into two major clusters (Cluster I & II) and one out-group (Cluster III). While, all Darjeeling cultivars were found clustered in cluster I (TH09, TH03, B668, T383, BS7/1A/76), cluster II was represented with majority of potential China tea cultivars collected from abandoned tea gardens. Further, popular tea cultivars from Assam (TTL01 and TTL02) were also found clustered in cluster II. Among the tea cultivars, TV02 and BGP138 exhibited with maximum genetic diversity (0.7), followed by S’stock01 and BGP138 (0.66), S’stock01 and BL9/376 (0.62). Larger leaf size of tea is considered desirable trait as it contributes towards higher photosynthetic efficiency, a key indicator of high yield potential in tea. Interestingly, tea cultivars with larger leaf size (BS13, BS05, BS64, BS40, Sidhbari01, Khalet05, BS06, BS47 and TV02) exhibited with genetic affinities and grouped together in cluster I. Likewise, high quality tea cultivars with ability to accumulate higher content of ECG (Epicatechingallate), EGCG (Epigallocatechin-3-gallate), EC (Epicatechin), Catechin and astringency factors including TTL2, T383, BL9/3/76, BGP126, BGP31, BS93, BGP144, BS107, BS34, BGP67, Mahalpat02, BGP17, BGP123, BGP72, BGP119, S’stock07 and Khilpat5 were grouped together in cluster II. Nevertheless, few of the tea cultivars with larger leaf size and high quality were also found highly heterogeneous (TV02, T383, BGP138, BS40, Khilpat05 and Mpat02) and were randomly intermixed in all three clusters, can be potential parental groups for breeding and genetic improvement of quality and yield traits in tea (Fig. 6).

### Suitability of TTFMS markers for genetic mapping

SSR markers have been preferred markers for genome mapping studies and dissection of complex traits. Successful utilization and testing of 589 polymorphic TF-SSR markers between two parental lines (P_1_ and P_2_) and respective bi-parental F_1_ populations (10 individuals) revealed five segregating patterns in 265 TF markers. Of these, 185 markers representing hk x hk (77), lm x ll (49), nn x np (47), ab x cd (9) and ef x eg (3) segregating patterns, can be futuristically utilised for construction of genetic map and establishing marker-trait association of targeted traits in tea (Supplementary file 4).

### Potential of codon reiteration in TFs of Tea

SSRs repeats in coding region contributes to repetitive pattern in protein sequences as tandem tri- and hexa-nucleotide SSR repeats leads to single amino acid repeats (SAARs). SAARs or codon reiteration is a unique mechanism which increases the size of protein due to reiteration of some codons more than others. Current data stipulates high abundance of tri-nucleotide repeats in CDS region, potentially codes for serine (19%) followed by glycine (11%), leucine (11%), aspartic acid (10%), threonine (10%), glutamine (10%), glutamate (10%), proline (10%) and histidine (9%), and least abundance of tyrosine (1%) in the TFs genes (Table S1). Of these, serine and glycine were considered to be most favoured amino acids (AAs) of a polypeptide. Nevertheless, hydrophobic proline and leucine were also found abundant transcripts encoding TF genes in tea^36^. Furthermore, AAs frequency of reiteration in coding region of TF genes varied from five to ten and eleven to twenty were also identified (Fig. S3a & b). Leucine, one of the most abundant AA was also found the most frequently reiterated in TFs of tea. Total 42 transcripts were identified in which near a reiterant, a second reiterant was retrieved in coding region. However, inherent interruption of SSR repeats due to mutations, few tri-nucleotide repeat encoding for histidine and glycine exhibited interruption possibly due to mutation over time in tea^37^ (Fig. S4a–d).

## Discussion

Simple sequence repeats (SSRs), a third major category of variations after CNVs and SNPs, are having important function in controlling long range interaction and genome packaging^3,38–41^. Being bias towards expansion than contraction, SSRs are considered as an important turning knob of evolution, and genome level structural and functional variability. Tea, a widely consumed non-alcoholic beverage produced in more than 60 countries have recorded increasing trend in production and consumption, ensuring higher return to farmers^7,8^. Multiple desirable attributes and key regulatory role of transcription factors, SSR markers derived from TFs can be potentially utilized for trait dissection and implementation of marker-assisted breeding in tea^5^. Furthermore, functionally relevant 403 core-set of TF SSR markers identified in this study can assist in high-throughput genotyping, authentication of various teas and large-scale fingerprinting studies. Therefore, enriching functionally relevant experimentally validated polymorphic TF derived SSR marker resource developed in the present study will expedite fingerprinting, genome mapping, linkage and diversity analysis efforts to assist in genetic improvement in tea^10,15,20–24,42^.

### Identification, distribution of SSRs in TF genes

Higher abundance of SSRs in transcripts encoding TFs (2,776 TF genes harbouring 3,687 SSR motifs) than other plant species possibly be associated with SSR search criteria and genomic attributes of targeted species^34^. Frequency of di-nucleotide repeats in TF genes is consistent with earlier reports of EST-SSR marker studies in tea, and other dicotyledon crops like *Actinidia eriantha*, *Luffaa egyptiaca*, *Paeonia*, *Amorphophallus*, *Colocasia esculenta*, *Rosa roxburghii*, and *Hevea brasiliensis*^8,13,43–50^. Furthermore, most frequent AG/CT repeats represent GAG, AGA, CUC and UCU codon encoding alanine and leucine exhibited with high abundance in protein sequences of tea and other plant species^10,51,52^. Contrarily, scarcity of GC repeat motifs in the data possibly be associated with less probability of CpG islands avoiding methylation mediated transcriptional interruptions^8^. Likewise, high abundance of tri-nucleotide motifs viz., AAG/CTT in TF genes were also reported to be predominant in dicotyledons^50^. The localisation of tri-nucleotide repeats in CDS region may be attributed to the fact that repeat length variations will not affect the reading frame of the protein. Likewise, di-nucleotide repeat abundance in untranslated regions (5'UTR) will not be affecting reading frame, hence, tolerated more in untranslated regions than CDS^10,51^. Moreover, variation in di-nucleotide repeats (GA/TC) present in 5'UTR has been correlated with important agronomic traits like amylose content in rice^53^. Interestingly, polymorphic di-nucleotide repeats TF-SSR markers belonging to functionally relevant TF families like B3, NAC, bHLH, C3H, and Myb-related localized to CDS regions were also assigned to various chromosomes of CSS tea genome. Furthermore, functionally relevant core-set of markers localized to UTRs and protein-coding regions can be potential markers for genetic analysis and establishing marker-trait association in tea^49^.

### GO classification and functional relevance of TF-SSRs

The GO enrichment analysis of TF genes harbouring SSRs depicted with high representation of GO terms like response to stimulus, response to abiotic stress, response to metabolic processes, cellular macromolecule biosynthesis and transcription, transcription regulator activity and transcription factor activity suggests the potential utility of current markers resource to identify trait-specific variations in tea. Furthermore, highly polymorphic core-set of TF-SSR markers identified in differentially expressed TF genes can be an important resource for eQTL analysis. Moreover, polymorphic SSR markers derived from bHLH (53), WRKY (37), C3H (32), Myb-related (31) and NAC (30) TF families reportedly involved in regulation of multiple enzymatic steps involved in quality related traits (flavonoids biosynthesis) are of utmost importance in targeted trait dissection^54^. Likewise, second most represented WRKY and NAC TFs harbouring highly polymorphic SSR markers were conferred to have key functional role in regulation of biotic^56–58^ and ABA mediated abiotic stress tolerance (cold and drought stresses) in tea^55,58,59^. Similarly, C3H and Myb-related TF families regulate dormancy status of vegetative buds^60^ and accumulation of anthocyanin pigment in tea, respectively^9^.

### Localization of TF-SSR markers

Polymorphism, expansion/contraction of SSR loci in CDS and untranslated regions (UTRs) of potential genes may lead to key variations influencing gain or loss of targeted traits^52,61^. Therefore, 589 polymorphic TF-SSR markers identified in this study are potential functionally relevant markers for trait dissection^62^. Furthermore, SSR polymorphism recorded in UTRs of TFs possibly be influencing the transcription/translation (5’UTR) and gene silencing (3’UTRs)^63^. Likewise, variations in CDS region might results in truncated protein formation^64,65^. Abundance of TFs harbouring short motifs in the transcribed region was also reported in many earlier studies^66–68^. The scarcity of longer microsatellites in TF genes might be due to the downward mutation bias and low persistence time^69^. Moreover, contraction mutation events happen more with increase in allele size due to which longer alleles tend to become shorter avoiding their infinite growth^65,70^. Therefore, the pattern of SSRs in TFs genes stipulates that tea genome possibly be under rapid evolution^71^.

### PPI network and functional significance

Protein–protein interaction is one of the important steps to mediate the action of expressed proteins to precisely regulate the signal transduction processes and homeostasis^5^. TFs, being key molecular players controlling gene expression of various growth and development processes undergo complex interactions with other proteins. Furthermore, variation in these proteins will have profound impact on other interacting proteins. In current study, direct significant interactions identified between the TF genes of tea harbouring polymorphic markers with volatile fatty acid biosynthesis, drought responsive, plant pathogen interactions and MAPK signalling pathways stipulates their putative functional consequences. Therefore, understanding the interactions of TFs harbouring polymorphic SSR markers will assist in rapid prediction of functional relevance in biological functions, and also have implication for QTLs analysis and marker assisted selection in tea^20^.

### Polymorphic potential, core marker selection, fingerprinting and genetic diversity analysis

Experimental validation of functionally relevant 862 markers with identification of 589 highly polymorphic and stable markers including 403 core-set TF-SSR markers can be utilised to study the impact of expansion/contraction repeats in targeted trait dissection (Table S4). Nevertheless, unsuccessful amplification in 20.2% TTFMS markers loci might be due to the insertion or deletion at primer binding sites of corresponding genomic sequences. Variations detected in UTRs and CDS regions may be correlated with regulation of gene function influencing quantitative and qualitative phenotypic variations in tea^27^. The 18 polymorphic functional domain associated TF-SSR markers may have utility for mapping of specific regulatory genes along with direct allele selection^43,44^ and its impact on comparative gene expression^46^. High polymorphic rate of novel TTFMS markers (589; 79.8%) including 403 core set of markers suggests wider utility in genetic analysis in tea^28^. Additionally, comparable mean gene diversity (*He*: 0.48) and polymorphic information content (PIC: 0.60) inferences also suggests importance of novel markers in various genotyping studies in tea^10,72–74^, similar to earlier studies in various crop plants like rice^4^, chickpea^31,32^ and sugarcane^5^. A subset of 15 informative polymorphic core set of TTFMS markers distinguishing 135 popular tea cultivars can be utilised futuristically as informative set of markers for larger scale fingerprinting studies^75^. Successful DNA fingerprinting application greatly depends on the various marker attributes including polymorphic potential, reproducibility and discrimination power. The high polymorphic potential (5.89 alleles/ per locus) detected with core set of TF –SSR markers was comparable to other studies^76,77^. Interestingly, high average PIC recorded with core-set of markers was significantly high as compare to earlier reports in tea^76–78^. Moreover, clustering of tea cultivars based phenotypic attributes (leaf characteristics) and biochemical parameters (ECG, EGCG, EC, Catechin and Caffeine) suggests their implications for selection of potential parental groups for breeding of high yielding quality tea cultivars^10,15,42,79^. Additionally, 185 TF-SSR markers with expected segregation patterns in tested bi-parental population can be directly utilized for genetic map construction and QTLs analysis in tea^13^.

### Expansion of codon repeats and their functional significance

Slippage mediated expansion and contraction of tri-nucleotide repeats (do not disturb the protein reading frame) are tolerated more in coding region. In current study, tri-nucleotide repeats were more abundant in the CDS region might be due to mutation pressure or possibly due to positive selection for specific amino acid repeats in the polypeptides encoding TF genes of tea^80^. Expansion of codon repeats encoding hydrophilic AAs Serine (≥ 14 repeats) indicates more tolerance than hydrophobic AAs in coding regions due to strong selection pressure eliminating basic and hydrophobic AAs repeats^81^. Further, two acidic (aspartic and glutamic acid), neutral (serine and threonine) and one basic (histidine) amino acid repeats found more reiterated due to tri-nucleotide repeat motifs, supports the abundance of polar and acidic AAs in TFs gene families of tea^82^. Leucine, among the most abundant and frequently reiterated AA in TFs genes in tea, suggests SSR dependent AA (leucine) reiteration which is predominantly reported in higher plant species^83^. Reiteration of single amino acid tandem tri/hexa-nucleotide repeat in various TF genes in dormancy (B3, C2H2 and MYB), secondary metabolite bio-synthesis (bHLH and MYB), abiotic stress response (ERF, NAC, GRAS, HSF, Tri-helix, WRK and bud and leaf pigmentation (TCP) suggest positive selection pressure for accumulation of these repeats and might have functional role in quality, yield and biotic & abiotic stress tolerance in tea^36,81^ (Fig. S6a–d).

## Conclusion

SSR repeats in regulatory genes influence the normal activity and function of the genes due to the repeat length (expansion and contraction) variation causing phenotypic changes in the plants. Due to limited availability of number of validated SSR markers from regulatory genes, identification of 1843 TF-SSR markers including 589 potential polymorphic markers will be a novel Tea Transcription Factor derived MicroSatellites (TTFMS) marker resources in tea. Furthermore, identification of 403 functionally relevant core-set of TF SSR markers with desirable marker attributes (Na: 3–17 per locus; He: 0.48; Ho: 0.73; PIC: up to 0.90) and successful extrapolation in diversity characterization of 135 tea popular cultivars suggests wider implications of novel marker resources. Additionally, appropriate segregating patterns of 185 markers in bi-parental mapping population representing hk × hk (77), lm × ll (49), nn × np (47), ab × cd (9) and ef × eg (3) stipulates their potential applications in genetic mapping and establishing marker-trait association in tea. Polymorphic core set of TF-SSR markers retrieved in bHLH, Myb-related, WRKY, C2H2, C3H, ERF, NAC, FAR1, G2-like and MYB suggests their key role in combining quality (flavonoid biosynthesis) and stress tolerance in high yielding tea cultivars. Key attributes including polymorphic potential, stability, functional relevance and genome-wide representation across all 15 chromosomes suggests wider implications of novel TF-SSR resource to accelerate molecular breeding efforts and traits dissection in tea.

## Methods

### Data utilised for mining of transcription factor (TFs) genes

Global in-house transcriptome sequence data of 20 diverse tea cultivars was utilized for mining of putative transcription factors derived SSR markers in this study {PRJNA306068^55^, SRR7037029, SRR7037030, SRR7037031, SRR7037032, SRR7037033, SRR7037034, SRR7037035^56^, PRJNA450985 and PRJNA520786^55^}.

### Plant material

All the tea cultivars utilized in the current study are maintained at CSIR-Institute of Himalayan Bioresource Technology, India, [Latitude (32^◦^6′ 52 N); Longitude (76^◦^33′ 24E); altitude of 5298 ft; mean annual rainfall 2493 mm; average annual temperature 19.1^◦^C]. The plant material was collected according to the institutional, national, and international guidelines and legislation.

### RNA Isolation and cDNA library construction

Twenty tea cultivars having inherent high quality, yield and tolerance to various abiotic and biotic stresses were utilised for transcriptome sequencing. Leaf tissue sample were frozen in liquid nitrogen and stored at (−) 80 °C for RNA isolation^84^. Furthermore, mRNA isolation was carried out using *iRIS* protocol and quantity/quality of RNA was analysed using Nanodrop2000 and Agilent Bio-analyzer Chip RNA 7500 series II. For cDNA library preparation illumine TruSeq RNA Sample Prep Kit v2 LS (Illumina Inc., San Diego, CA) was utilised and libraries of 10 pM concentration were loaded on the flowcell for Paired End (PE) sequencing.

### De novo assembly and functional annotation

For base calling and de-multiplexing of generated data, Illumina Cassava 1.8.2 pipeline (http://support.illumina.com/) was utilised followed by various quality control steps using NGS QC tool kit^78,87^. Trinity Software package ver2.3.2 used for *de novo* transcriptome assembly of high quality reads with minimum cut-off length of 300 bp. Homology based putative functional annotation of assembled sequences was performed using publically available Plant Transcription Factor database (http://planttfdb.cbi.pku.edu.cn/) with an e-value of ≤ 1e-5. The gene ontology annotation was performed using Blast2GO and WEGO tools^85^. Furthermore, assembled transcripts encoding transcription factor genes of tea were successfully mapped to the 15 chromosomes of reference tea genome^28^.

### TTFMS and TTFDMS marker development

Assembled transcripts encoding transcription factors (TFs) were searched individually for microsatellites using MISA (http://pgrc.ipk-gatersleben.de/misa) and were characterised as perfect repeats (di-nucleotide to hexa-nucleotide repeats) and hypervariable on the basis of repeat length^20^. NCBI open reading frame finder (ORF) (http://www.ncbi.nlm.nih.gov/projects/gorf) was used to identify the longest ORF in the transcripts. Further, amino acid sequence of predicted ORF was analysed using Pfam and BLAST searched against NCBI conserved domain and nr protein database (http://www.ncbi.nlm.nih.gov/Structure/cdd/wrpsb.cgi) to identify putative conserved domain. SSR search criteria was kept six repeats for di-nucleotide repeats, five for tri-, tetra-, penta- and hexa-nucleotide repeats, and compound microsatellites were defined with two repeats interrupted by ≤ 100 bp. Forward and reverse primers from flanking region of SSR loci were designed using Primer 3 tool (Parameter; 18-23 bp primer length, 40–60% GC content, 55–60 °C melting temperature and product size between 100 and 350 bp). Further, for codon reiteration analysis only tri-/hexa-nucleotide repeats were targeted in the TFs genes of Tea. The tri-/hexa-nucleotide repeats encoding for amino acid ≥ 5 were identified and were encoded as single amino acid repeats in the TFs genes of Tea and every uninterrupted single amino acid repeats was considered as a unique event in the transcript^36^.

### PPI Network analysis of TFs harbouring SSR

The PPI network for TF genes harbouring SSR were built utilising STRING PPI network of Arabidopsis (https://string-db.org/)^86^. Further the network was visualised using Cytoscape v3.4. Further, correlation between the TF genes was determined on the basis of significant correlation edges with its TAIR orthologs.

### DNA isolation, PCR amplification and data analysis

Young green leaves were utilised for genomic DNA isolation from random cultivars representing three traditional varietal types namely [Assam (*C. assamica*), China (*C. sinensis*) and Cambod/Indian type (C. *assamica* spp. *lasiocalyx*)] for screening of TF-SSR markers (Table S2). Further, genomic DNA of 135 tea cultivars and 10 individuals of F1 mapping population along with parental lines were isolated using DNeasy Plant Mini Kit (Qiagen, Germany) to predict functional diversity and for genetic mapping analysis (Table S3). Quantity and quality of DNA was analysed using NanoDrop 2000 OD_260_/OD_280_ (Thermo Scientific, Lithuania) and integrity with 0.8% agarose gel. PCR amplification was performed using 25 ng of genomic DNA and amplified products was separated on denaturing polyacrylamide gels containing 7% of polyacrylamide and 7 M urea in 1 × TBE buffer. Denatured product was loaded on to the gel Sequi-Gen GT system (Bio-Rad, Australia) and size was measured using 50 bp ladder standard^10^. SSRs alleles were scored in binary format 0 (absent)/1 (present) and were utilised for genetic relationship determination and estimation of marker amplification frequency and polymorphism potential in tea cultivars. The observed heterozygosity (*Ho*), expected heterozygosity (*He*) and polymorphism information content (*PIC*) was estimated using power marker software version 3^87,88^. Further, the dendrogram was constructed on the basis of Nei’s genetic distance matrix using neighbour-joining (NJ) methodology with 1000 bootstrap replicates^28,50,85,89,90^. Further, for genetic mapping analysis, tea being a cross pollinated plant species, four alleles representing five different segregation patterns viz.; hk × hk, lm × ll, nn × np, ab × cd and ef × eg were utilised^13^. Core set of TF SSR markers were identified using *PI* and *PIsibs* statistics for individual marker using GenAlEx version 6.5^22,91^. Further, additional parameters including PIC (≥ 0.5) and alleles (≥ 3 alleles/ loci) were also considered for identification of core set of TF-SSR markers^22^.

## Supplementary Information

Supplementary Information 1.

Supplementary Information 2.

Supplementary Information 3.

Supplementary Information 4.

Supplementary Information 5.
