# Supplementary material for: Genome-wide identification and characterization of functionally relevant microsatellite markers from transcription factor genes of Tea (Camellia sinensis (L.) O. Kuntze)
Source: Sci Rep. 2022 Jan 7;12:201. doi: 10.1038/s41598-021-03848-x (PMC8742041; doi:10.1038/s41598-021-03848-x)
Supplement: Supplementary file 1 — Supplementary Information 1. [file 41598_2021_3848_MOESM1_ESM.pdf]

Supplementary Information

Genome-wide identification and characterization of functionally relevant microsatellite markers from transcription factor genes of Tea (*Camellia sinensis* (L.) O. Kuntze)

Rajni Parmar[1,2], Romit Seth[1], Ram Kumar Sharma[1,2]*

[1]Biotechnology Department, CSIR-Institute of Himalayan Bioresource Technology (CSIR-IHBT), Palampur, Himachal Pradesh, 176061, India

[2]Academy of Scientific and Innovative Research (AcSIR), CSIR-HRDC Campus, Ghaziabad, Uttar Pradesh- 201 002, India

***Correspondence:**
Dr Ram Kumar Sharma
Biotechnology Department
CSIR-Institute of Himalayan Bioresource Technology
Palampur (H.P)
India
Email: rksharma.ihbt@gmail.com

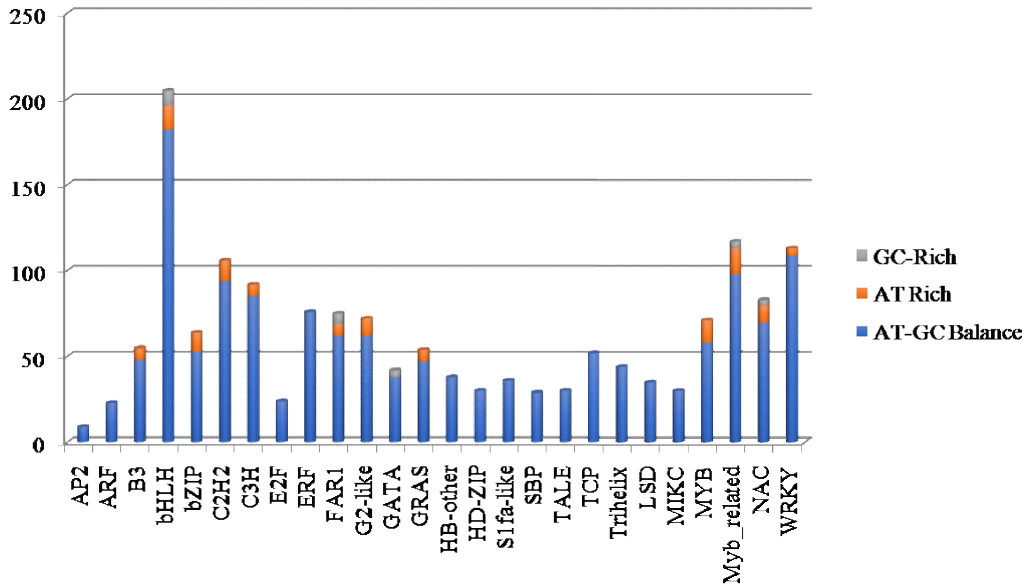

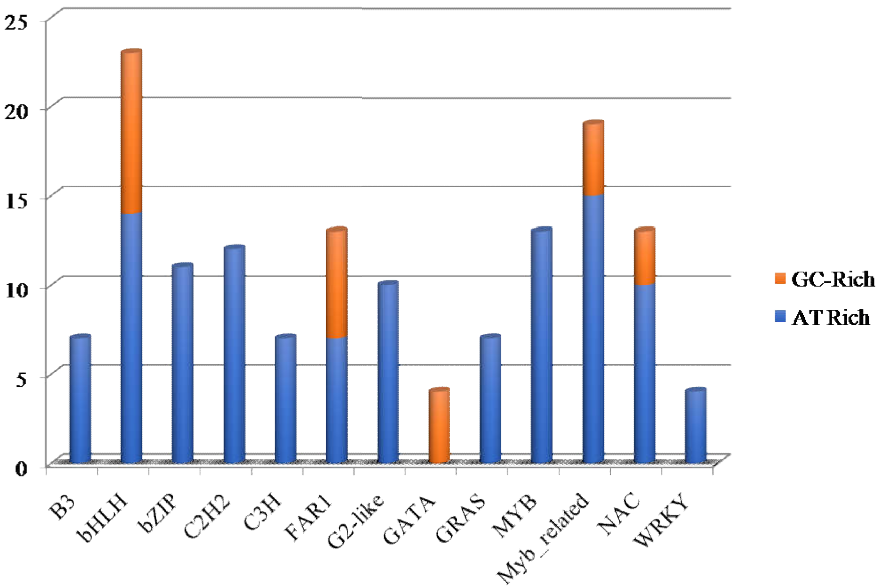

Fig. S1: Distribution by family in terms of motif nucleotide base composition

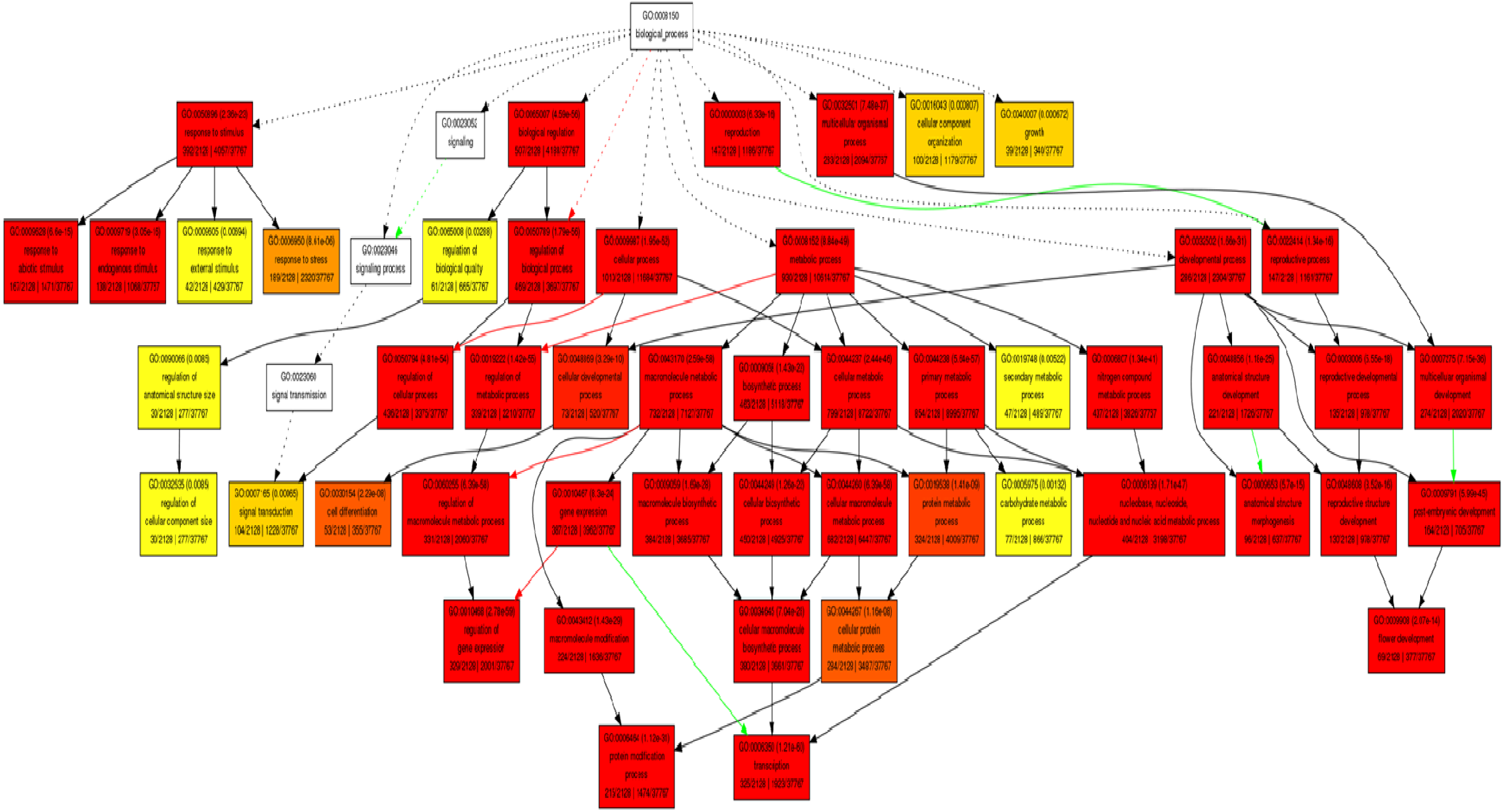

(a)

Fig. S2 Gene Ontology (GO) enrichment analysis **(a)** Biological Processes

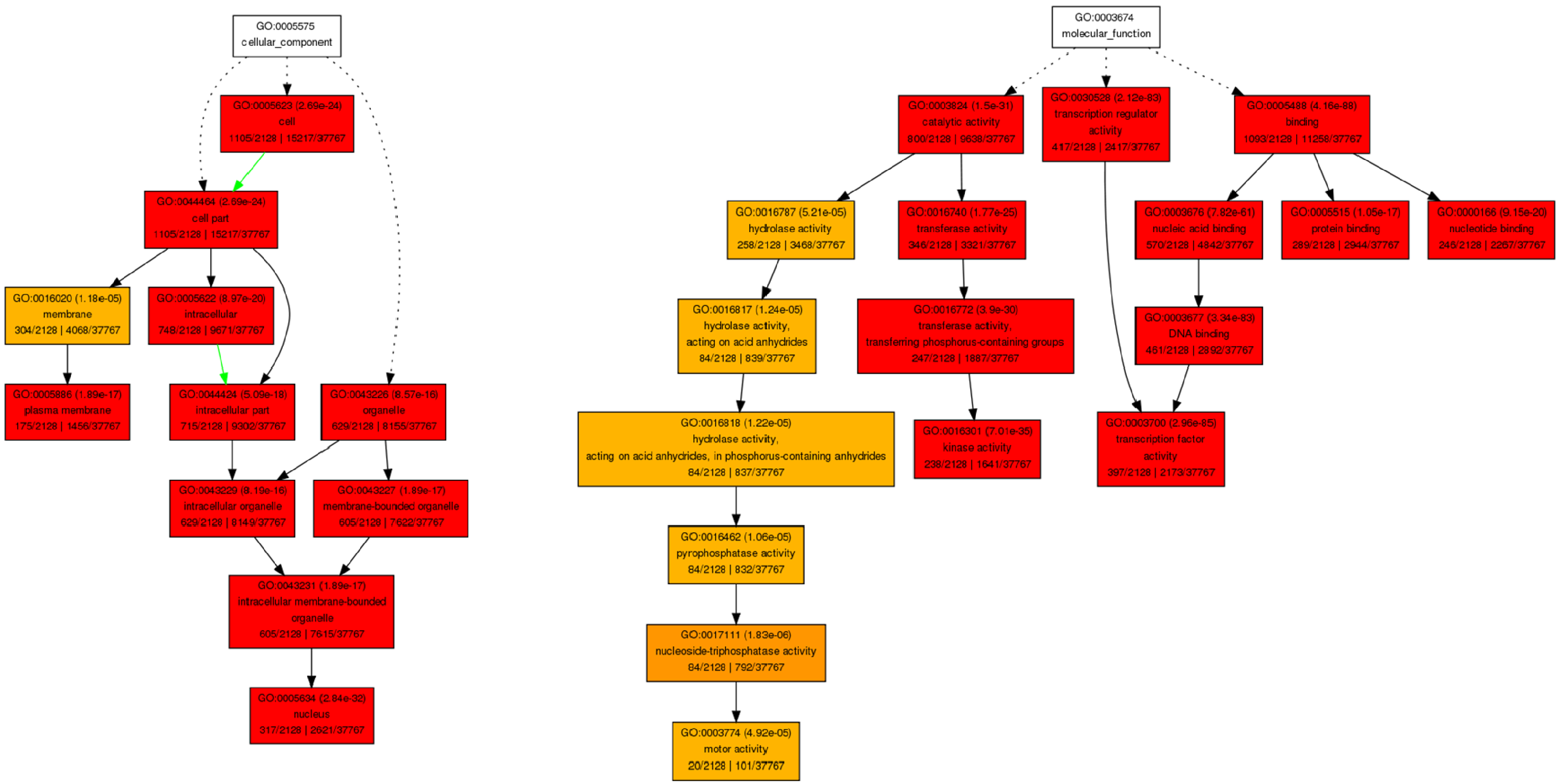

(b) (c)

Fig. S2 Gene Ontology (GO) enrichment analysis **(b)** Cellular Component and **(c)** Molecular Function

S.No.	Type of Amino Acid	Symbol	Amino Acid	Symbol	Number
**1**	Non-Polar, Aliphatic R Groups	Gly	Glycine	G	46
**2**	Non-Polar, Aliphatic R Groups	Ala	Alanine	A	21
**3**	Non-Polar, Aliphatic R Groups	Pro	Proline	P	41
**4**	Non-Polar, Aliphatic R Groups	Val	Valine	V	29
**5**	Non-Polar, Aliphatic R Groups	Leu	Leucine	L	45
**6**	Non-Polar, Aliphatic R Groups	Ile	Isoleucine	I	15
**7**	Non-Polar, Aliphatic R Groups	Met	Methionine	M	10
**8**	Aromatic R Groups	Phe	Phenylalanine	F	17
**9**	Aromatic R Groups	Tyr	Tyrosine	Y	4
**10**	Aromatic R Groups	Trp	Tryptophan	W	10
**11**	Positively Charged R Groups	Lys	Lysine	K	25
**12**	Positively Charged R Groups	Arg	Arginine	R	29
**13**	Positively Charged R Groups	His	Histidine	H	36
**14**	Polar Uncharged R Groups	Ser	Serine	S	81
**15**	Polar Uncharged R Groups	Thr	Threonine	T	43
**16**	Polar Uncharged R Groups	Cys	Cysteine	C	13
**17**	Polar Uncharged R Groups	Asn	Asparagine	N	18
**18**	Polar Uncharged R Groups	Gln	Glutamine	Q	42
**19**	Negatively Charged R Groups	Asp	Aspartic Acid	D	44
**20**	Negatively Charged R Groups	Glu	Glutamate	E	42

Table S1: Potential codon reiterated in putative TF genes of Tea.

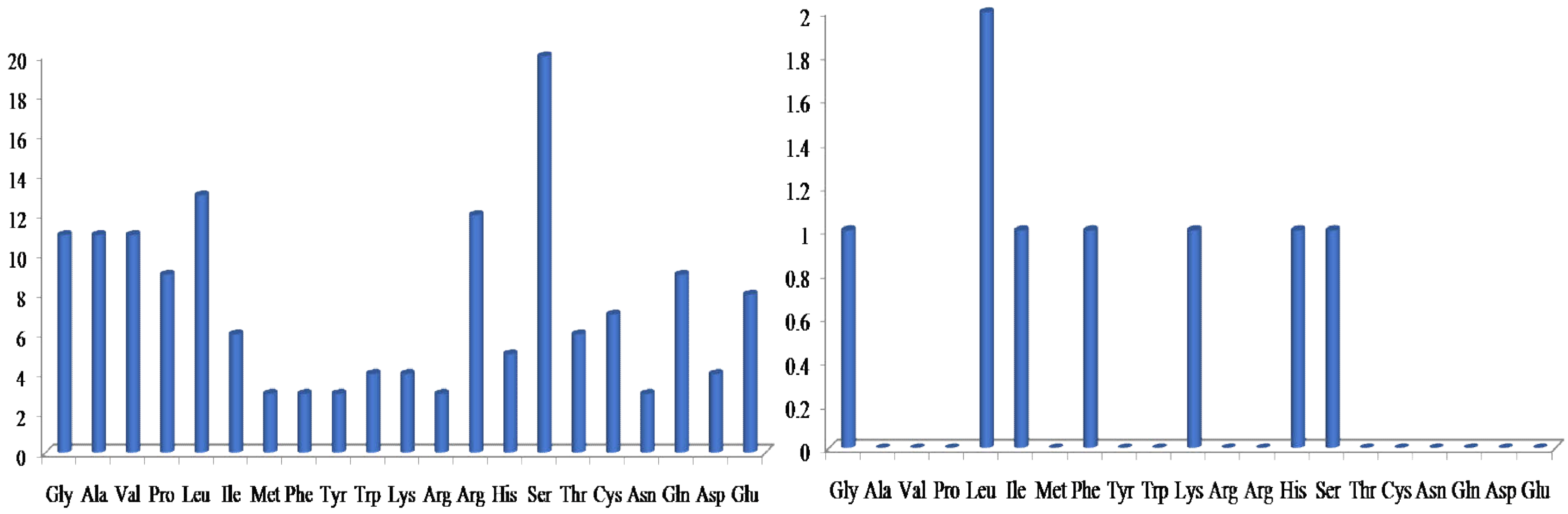

(a) **(b)**

Fig. S3 Amino acid reiterants in proteins **(a)** Frequency of reiterants of five to ten identical amino acids residues. **(b)** Frequency of reiterants of eleven to twenty identical amino acids residues.

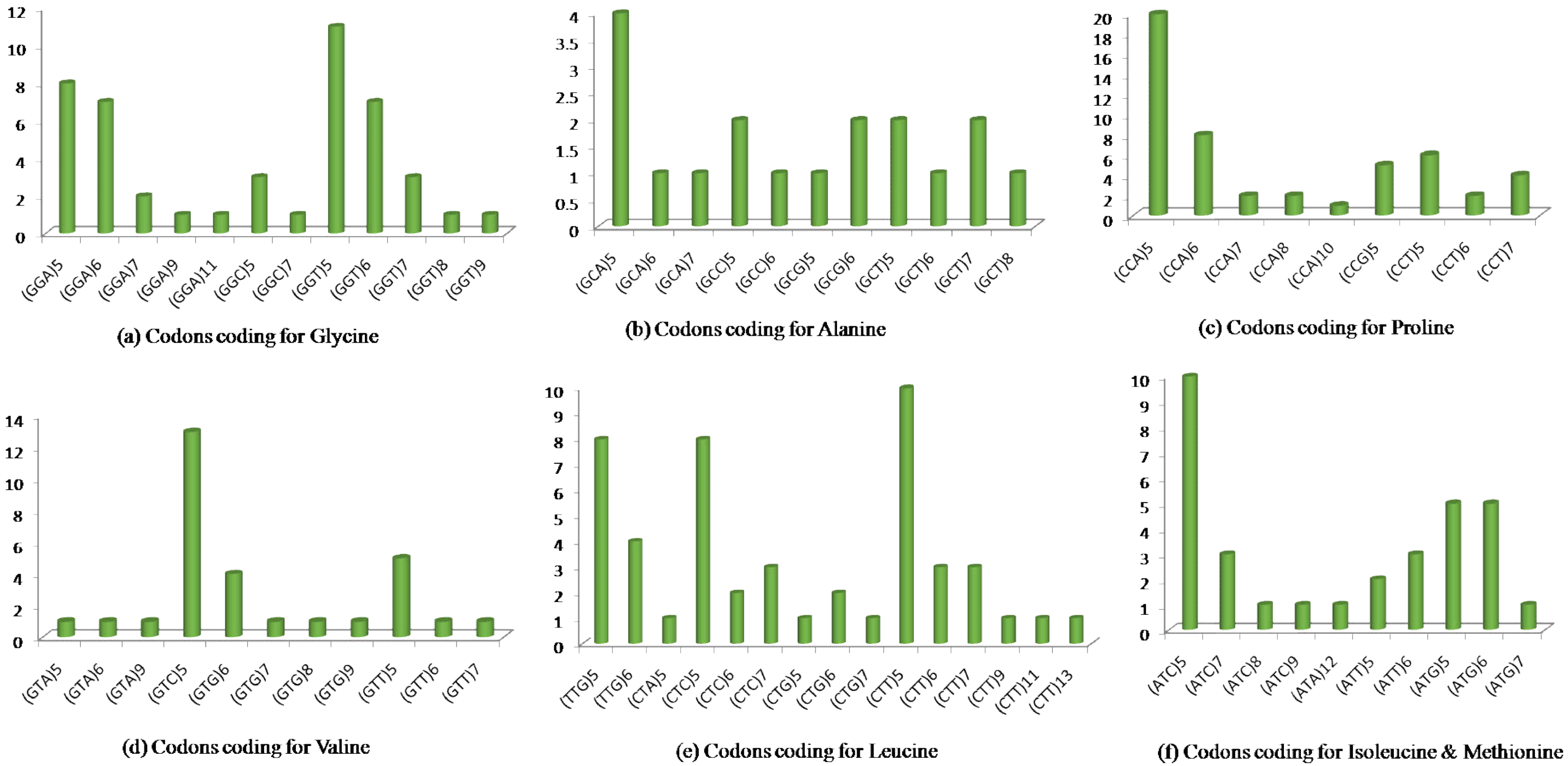

Fig. S4a Codon reiterants (Non-Polar, Aliphatic R Groups)

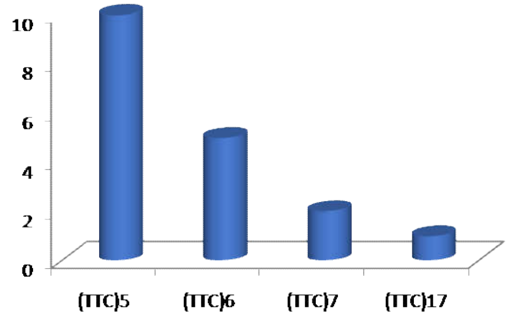

(a) Codons coding for Phenylalanine

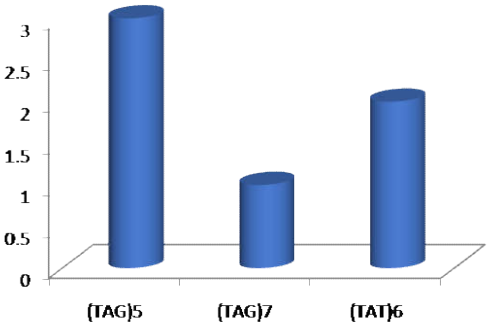

(b) Codons coding for Tyrosine

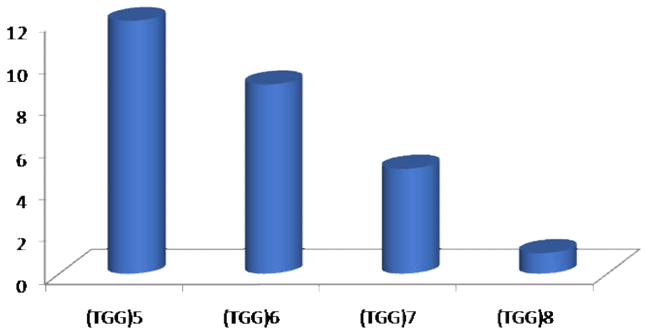

(c) Codons coding for Tryptophan

Positively Charged R Groups

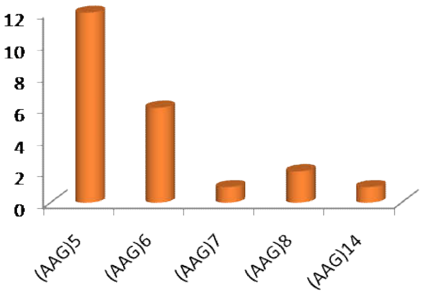

(a) Codons coding for Lysine

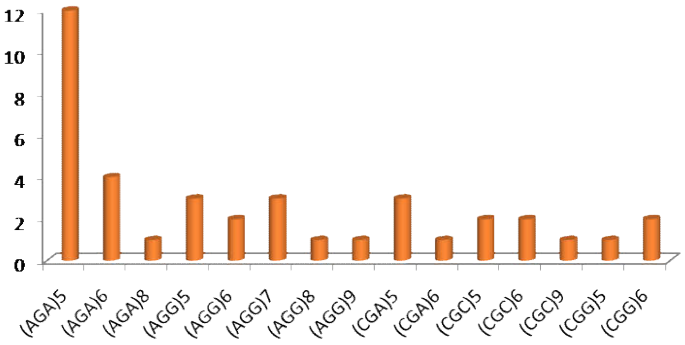

(b) Codons coding for Arginine

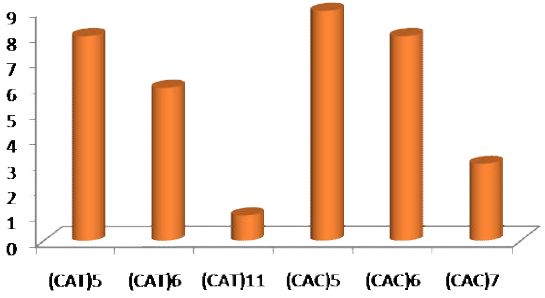

(b) Codons coding for Histidine

Fig. S4b Codon reiterants (Aromatic R Groups)

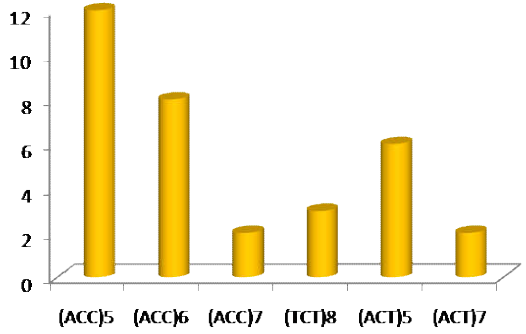

(a) Codons coding for Threonine

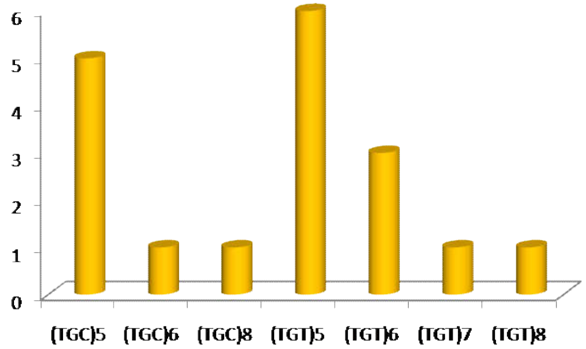

(b) Codons coding for Cysteine

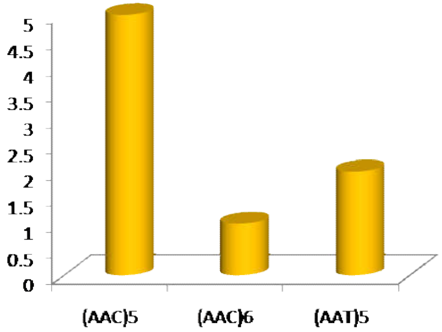

(c) Codons coding for Asparagine

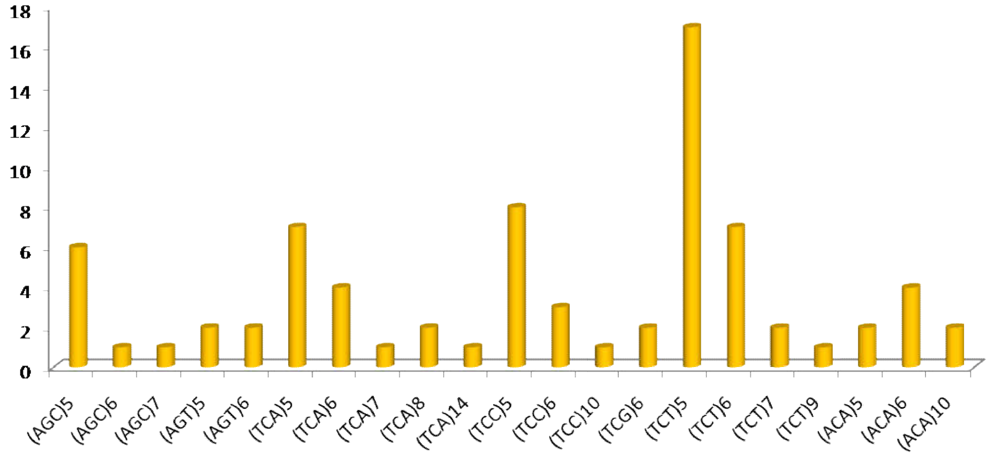

(d) Codons coding for Serine

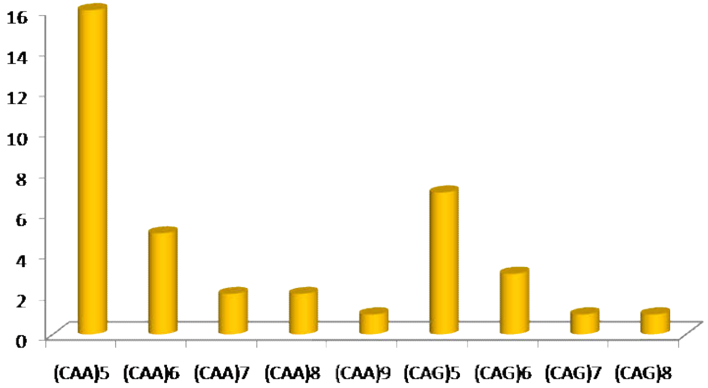

(d) Codons coding for Glutamine

Fig. S4c Codon reiterants (Polar Uncharged R Groups)

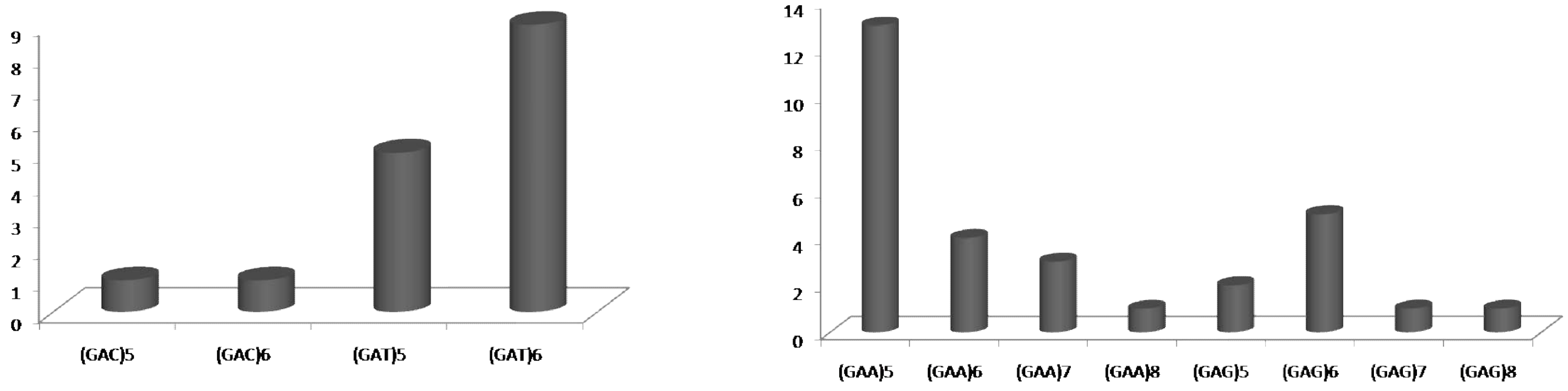

(a) Codons coding for Aspartic Acid

(b) Codons coding for Glutamate

Fig. S4d Codon reiterants (Polar-charged R Groups)

Table S2: List of eight genotypes utilized for screening of TF-SSR markers.

S.No.	Genotypes	Varietal Type	Remarks
**1**	TV 1	Assam	Average yield and Quality, Moderately Susceptible to Drought
**2**	TV 19	Cambod	High Yield and Quality, Drought Tolerant
**3**	SA 6	Assam	Blister Blight Tolerant, Avg quality and yield
**4**	ASHA	China	Blister Blight Sensitive, Quality
**5**	TS 449	Assam	High Quality But Average Yield, Drought Tolerant
**6**	C 6017	Cambod	Susceptible to Drought
**7**	TEENALI	Assam	_
**8**	U 9	Assam	High Yielding, Moderately resistant to blister blight

Table S3: List of 135 genotypes utilized for functional genetic diversity characterization using TF-SSR markers.

S. No	Name	Type
**1**	BS-13	China
**2**	BS-06 (12)	China
**3**	BS-40	China
**4**	TH-9 (156)	China
**5**	BS-05	China
**6**	SIDHBARI-01	China
**7**	BS-47 (42)	China
**8**	KHALET-05	China
**9**	UPASI-09	Assam
**10**	S'stock-01	Assam
**11**	TTL-1	Assam
**12**	BS-64	China
**13**	BB-668 (5)	China
**14**	BS-09 (16)	China
**15**	TV-2	Assam
**16**	TTL-2	Assam
**17**	T-383 (152)	China
**18**	BL/9/3/76 (7)	China
**19**	KHALAG-02	China
**20**	BS-66	China
**21**	BS-81	China
**22**	MANSIMBAL-07	China
**23**	BS-19 (24)	China
**24**	BS-91	China
**25**	BS-22 (26)	China
**26**	BS-31	China
**27**	MAHALPAT-05	China
**28**	BS-69	China
**29**	MAHALPAT 7	China
**30**	BS-77 (69)	China
**31**	BGP-86	China
**32**	BS-105	China
**33**	BS-79	China
**34**	BS-89	China
**35**	BGP-146	China
**36**	BS-96	China
**37**	BS-110	China
**38**	CPF-01	China
**39**	BGP-122	China
**40**	BS-12 (18)	China
**41**	BGP-69	China
**42**	BS-23	China

**43**	BGP-66	China
**44**	SULAH-02	China
**45**	MANSIMBAL-10	China
**46**	BGP-118	China
**47**	BGP-133	China
**48**	BGP-73	China
**49**	BGP-137	China
**50**	BGP-157	China
**51**	KANGRA-JAT	China
**52**	CEF-02	China
**53**	BS-98	China
**54**	BGP-126	China
**55**	BGP-31	China
**56**	BS-93	China
**57**	BGP-144	China
**58**	BS-107	China
**59**	BS-34	China
**60**	BGP-67	China
**61**	MAHALPAT 2	China
**62**	BS-07	China
**63**	SIDHBARI-05	China
**64**	BS-48	China
**65**	BS-11	China
**66**	LAHLA-01	China
**67**	BS-70	China
**68**	MANSIMBAL-17	China
**69**	MANSIMBAL-12	China
**70**	BS-76	China
**71**	BS-67	China
**72**	BAIJNATH-01	China
**73**	SIDHBARI-02	China
**74**	BS-108	China
**75**	BGP-28	China
**76**	CEF-03	China
**77**	BS-58	China
**78**	BS-56	China
**79**	BGP-19	China
**80**	CEF-01	China
**81**	BS-16	China
**82**	RAIPUR-04	China
**83**	BGP-17	China
**84**	BGP-123	China
**85**	BGP-72	China
**86**	BGP-119	China
**87**	KHALAG-04	China
**88**	BS-95 (81)	China

**89**	TH-3	China
**90**	BHATTU-22	China
**91**	BS-92	China
**92**	BS-29	China
**93**	BGP-63	China
**94**	HV-39	China
**95**	MANSIMBAL-08	China
**96**	BGP-138	China
**97**	BS-55 (50)	China
**98**	BS-49 (44)	China
**99**	BS-50	China
**100**	S'stock-09	Assam
**101**	BS-86	China
**102**	BS-24	China
**103**	BS-60	China
**104**	BS-102	China
**105**	BS-71	China
**106**	BS-99	China
**107**	S'stock-07	Assam
**108**	KHILPAT-5	China
**109**	BS-90	China
**110**	BS-80	China
**111**	BS-42	China
**112**	BS-02	China
**113**	BS-74	China
**114**	BS-85	China
**115**	Bs-106	China
**116**	BS-37	China
**117**	BS-18	China
**118**	BS-52	China
**119**	BS-104	China
**120**	BS-08	China
**121**	BS-43	China
**122**	PATTA-01	China
**123**	BS-44	China
**124**	BS-75	China
**125**	BS-21	China
**126**	BS-62	China
**127**	S'stock-05	Assam
**128**	BGP-121	China
**129**	BS-109	China
**130**	BGP-127	China
**131**	BGP-68	China
**132**	SULAH-I	China
**133**	BS-103	China
**134**	BS-03	China

135	KANGRA ASHA	China

Table S4: List of 26 polymorphic SSR markers from potential transcription factor families along with their validated functional role in various traits of tea.

S.No.	Locus Name	Repeat Type	Size	Alleles	TF Family	Putative Function in Tea	Reference
1	TTFDMS_13998	(AGG)5	140:240	5	B3	Dormancy status of bud in Tea	Thirugnanasambantham et al., 2013
2	TTFDMS_9683	(GA)17	250:300	5	BBR-BPC		
3	TTFMS_10094	(AT)6	160:200	2	bHLH	Secondary metabolism of Tea	Zhao et al., 2012
4	TTFDMS_67364	(TGG)5	170:180	2	bHLH		
5	TTFMS_1538_A	(CAC)5	150:170	2	bHLH		
6	TTFMS_22978	(CT)8	200:600	9	bHLH		
7	TTFMS_1011	(TA)6	200:250	2	bHLH		
8	TTFMS_17320	(AG)8	150:200	6	C2H2	Dormancy status of bud in Tea	Thirugnanasambantham et al., 2013
9	TTFDMS_4764	(CAC)5	300:400	1	ERF	Response against abnormal temperature stress	Wu et al., 2015
10	TTFDMS_66493	(GTG)6	150:250	9	FAR1		
11	TTFMS_59623	(AG)6	400:500	2	FAR1		
12	TTFDMS_26427	(TC)8	200:500	11	FAR1		

13	TTFDMS_36100	(AAG)5	250:400	7	G2-like		
14	TTFMS_25851_A	(CTG)5	150:200	1	GRAS	Response to abiotic stresses and Gibberelic acid treatment in tea (Salt Stress)	Wang et al., 2018
15	TTFDMS_299	(CT)9	400:500	3	HSF	Regulation under temperature stress	Liu et al., 2016
16	TTFMS_1264	(TC)8	150:300	5	LBD		
17	TTFDMS_790	(CT)9	700:800	2	MYB	Dormancy and CsMYB4a is negatively correlated to accumulation of six flavan-3-ols and other phenolic acids	Li et al., 2017 & Thirugnanasambantham et al., 2013
18	TTFDMS_13383	(AT)6	230:250	1	NAC	Accumulate stress-inducible genes or metabolites and enhances stress tolerance (Blister Blight in Tea)	Wang et al., 2016 &Jayaswall et al., 2016
19	TTFDMS_25118	(TCT)6	150:370	4	Nin-like	Bud and Leaf Colour Development	Wei et al., 2016
20	TTFDMS_17254	(CT)8	200:450	4	Nin-like		
21	TTFDMS_23789	(CAC)6	70:150	4	TCP	Leaf Development and Hormonal stimuli in Tea	Wu et al., 2017

22	TTFDMS_39438	(CT)6	230-250	2	Trihelix	Response to abiotic stresses in tea (Salt Stress)	Li et al., 2017
23	TTFDMS_7051	(CAA)5	250:300	1	Trihelix		
24	TTFMS_63882_B	(AG)6	150:250	3	Trihelix		
25	TTFMS_1910_A	(TC)7	220:250	2	WRKY	Responses to extreme temperatures (Cold and Drought stress) controlling ABA signalling, Plant immunity	Jayaswall et al., 2016, Wang et al., 2016 & Wu et al., 2016
26	TTFMS_1593	(CAA)5	200:300	7	WRKY		
